# Supplementary material for: Splicing factor BUD31 promotes ovarian cancer progression through sustaining the expression of anti-apoptotic BCL2L12
Source: Nat Commun. 2022 Oct 21;13:6246. doi: 10.1038/s41467-022-34042-w (PMC9587234; doi:10.1038/s41467-022-34042-w)
Supplement: Supplementary file 1 — Supplementary Information [file 41467_2022_34042_MOESM1_ESM.pdf]

Supplementary Information

Supplementary Figures

Figure S1, related to Figure 1

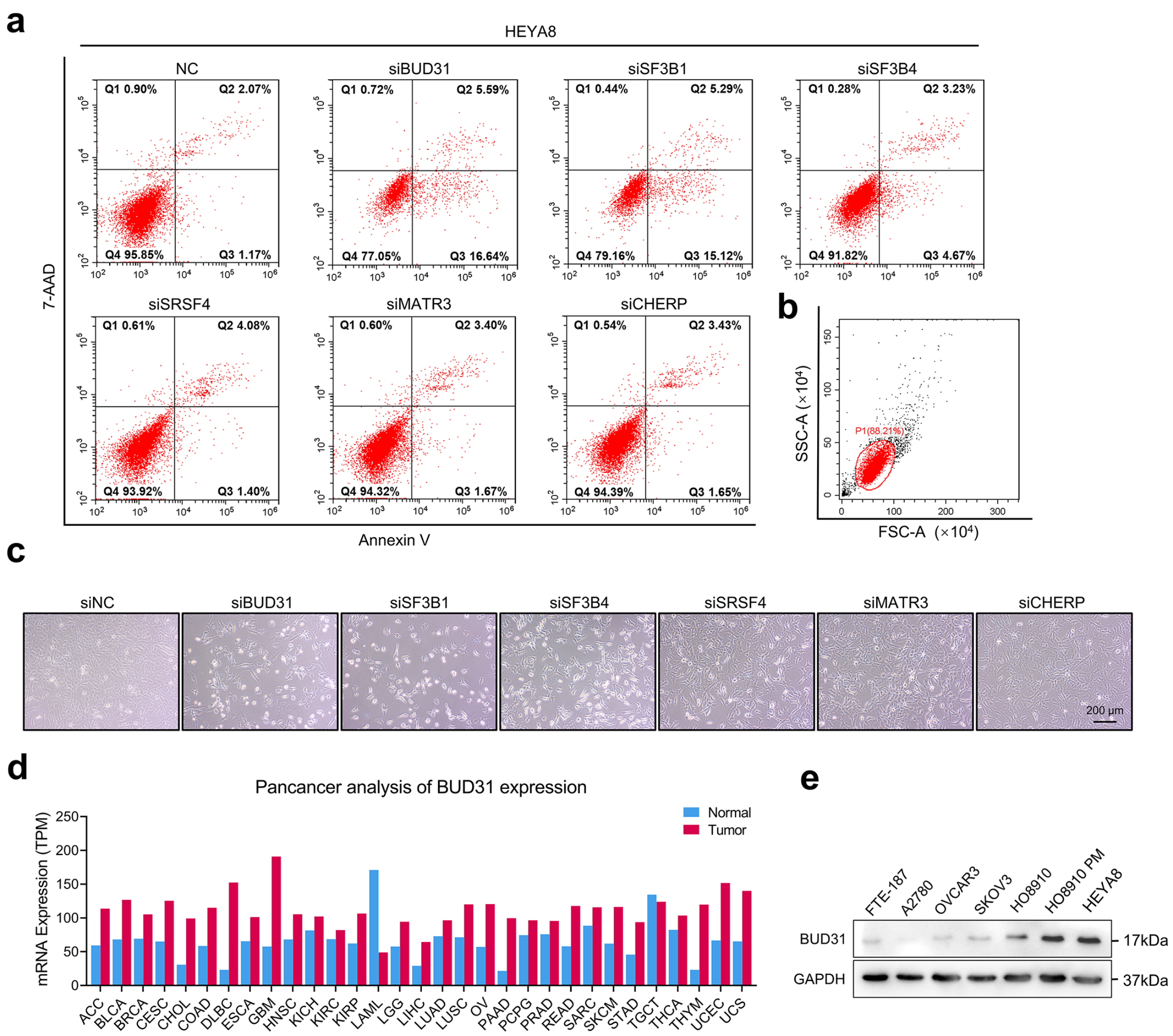

Figure S1. RNAi screen of six splicing factors and BUD31 expression across all TCGA tumors and ovarian cancer cell lines, related to Figure 1. (a) Flow cytometry assay was performed to determine the apoptosis cells percentage after siBUD31, siSF3B1, siSF3B4, siSRSF4, siMATR3,

and siCHERP treatment. **(b)** Representative gating strategy of flow cytometry in detecting apoptosis cells. **(c)** Representative light microscopy photos of HEYA8 cells treated with siBUD31, siSF3B1, siSF3B4, siSRSF4, siMATR3, and siCHERP. Scale bar, 200 μm. **(d)** Pan-cancer analysis of mRNA expression of *BUD31*. Expression profiles of tumor and normal samples were from TCGA and GTEx databases. **(e)** BUD31 protein expression in ovarian cancer cell lines compared with FTE187, an immortalized fallopian tube cell line. Source data are provided as a Source Data file.

Figure S2, related to Figure 2

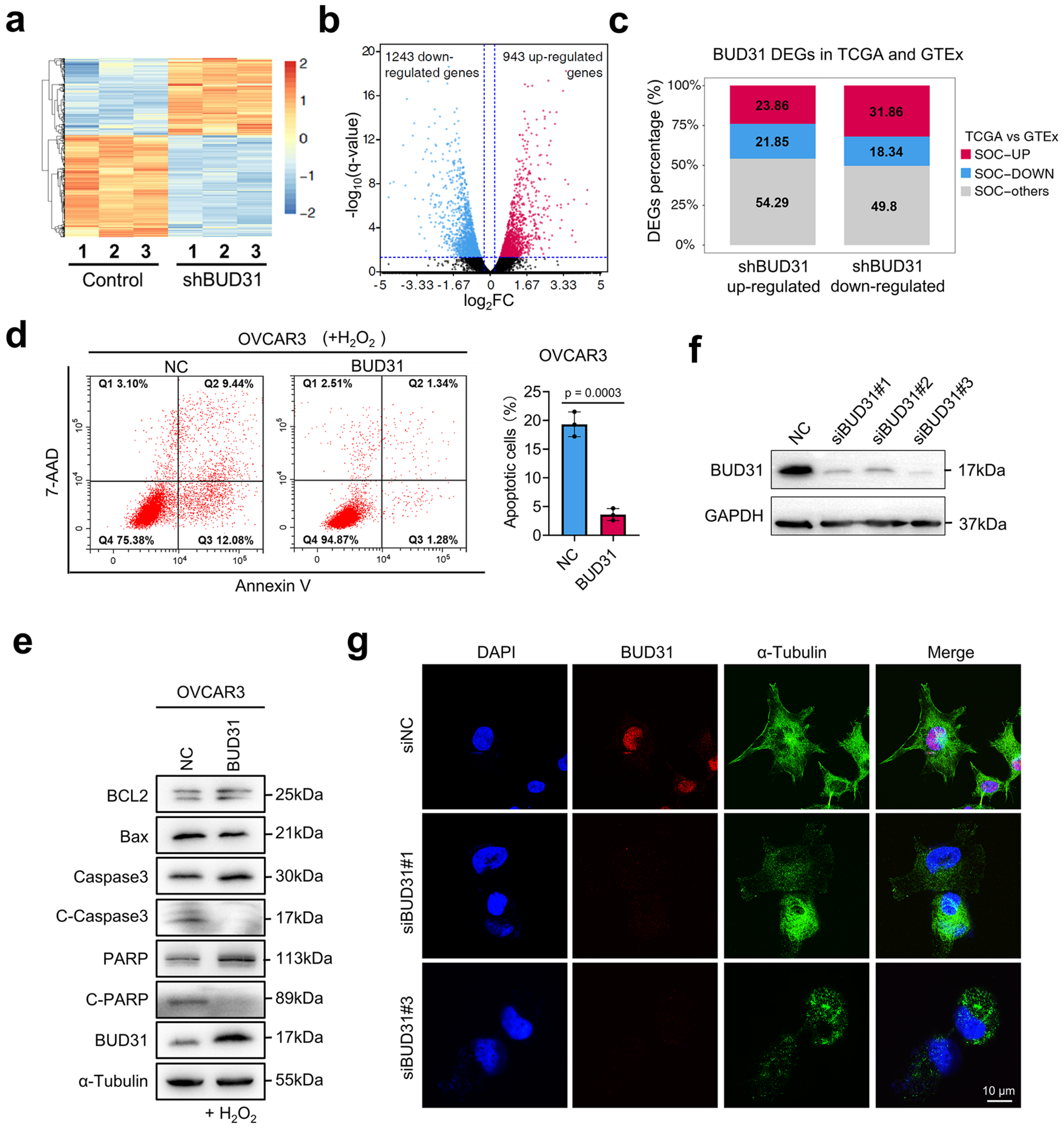

Figure. S2. Differentially expressed genes after BUD31 knockdown and BUD31 knockdown induced cell apoptosis, related to Figure 2. (a) Heatmap of the gene expression profile in HEYA8 cells knocking down BUD31 with corresponding control (n = 3 independent samples per group). **(b)** Volcano plot of differentially expressed genes (DEGs) in HEYA8 cells silencing BUD31 induced by doxycycline. The cutoff was set as q-value < 0.05 and fold change (FC) > 1.7 or < 0.6. 1243 genes were downregulated, and 943 genes were upregulated. **(c)** The gene expression of the DEGs in HGSOC samples (n = 374) in the TCGA database versus normal ovaries (n = 180) in the GTEx database. The percentages of the DEGs belonging to the high or low expression genes in HGSOC were shown. **(d)** Apoptotic cells staining with Annexin V/7-AAD were detected by flow cytometry in OVCAR3 cells with BUD31 overexpression (n = 3 biologically independent experiments). Apoptotic cells percentage included early and late apoptotic cells. Cells were treated with 400 μM H_2O_2 for 4 h before detection. **(e)** Apoptotic markers' expression was detected by western blot after BUD31 overexpression in OVCAR3 cells in the presence of H_2O_2. C-Caspase3, cleaved-caspase3. C-PARP, cleaved-PARP. **(f)** The efficiency of a siRNA suit silencing BUD31 was verified with western blot**. (g)** Tubulin structure and cell morphological changes were detected with high-speed confocal laser scanning. Two effective siRNA targeting BUD31 (siBUD31#1 and siBUD31#3) were used for silencing. Scale bar, 10 μm. P-value was obtained by unpaired two-tailed Student's t-test **(d)**, and the results are presented as the mean ± S.D. Source data are provided as a Source Data file.

Figure S3, related to Figure 3

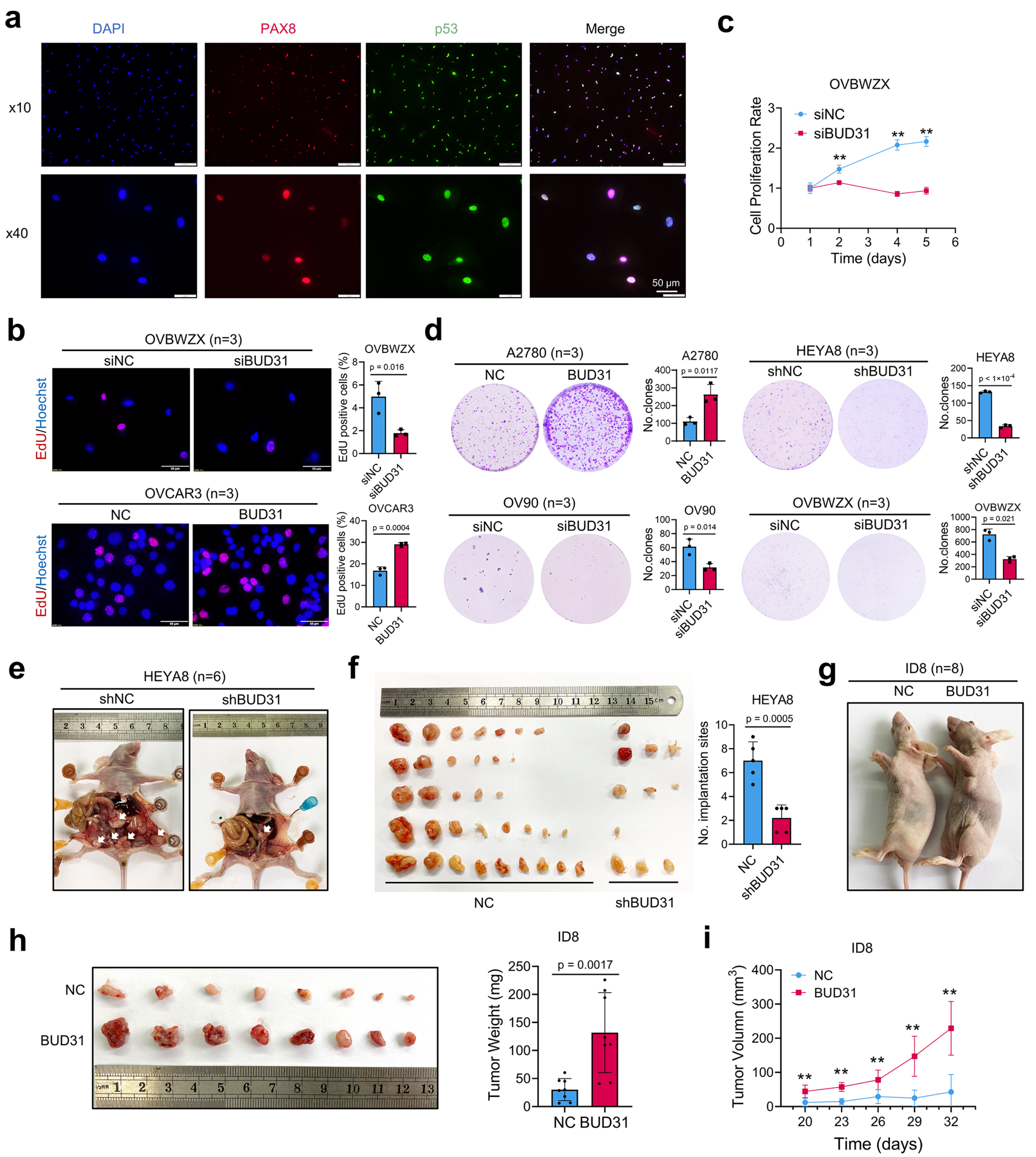

Figure. S3. Knockdown BUD31 inhibits ovarian cancer growth *in vitro* and *in vivo*, related to Figure 3. (a) Immunofluorescence stain of ovarian cancer marker PAX8 and p53 in OVBWZX cells derived from ovarian cancer patient's ascites. Scale bar, 50 μm. **(b)** EdU assay in primary ovarian cancer cells OVBWZX and OVCAR3 silencing BUD31 (n = 3 biologically independent experiments). Scale bar, 50 μm. **(c)** MTT assay was performed to determine the cell viability in OVBWZX cells knocking down BUD31 (n = 4 biologically independent experiments). **(d)** The clonogenic assay was performed in ovarian cancer cell lines with BUD31 overexpression (A2780 and OV90) or knockdown (HEYA8 and OVBWZX) compared to corresponding controls (n = 3 biologically independent experiments). **(e-f)** Xenograft tumors from intraperitoneal injection with HEYA8 cells silencing BUD31 or corresponding control **(e)** and the number of peritoneal implantation sites were counted (n = 5 mice per group when the mice were sacrificed, one pair dead). Representative images after exploratory laparotomy showed peritoneal implantation **(f). (g-i)** Representative images of xenograft nude mice **(g)**, xenograft tumor weight **(h),** and volume **(i)** in mice subcutaneously injected with ID8 cells with BUD31 overexpression compared to the corresponding control (n = 8 mice per group). P-value was obtained by unpaired Student's t-test, two-tailed **(b, c, d, f, h, i)**, and the results are presented as the mean ± S.D. $*p < 0.05$, $**p < 0.01$

Figure S4, related to Figure 4

a

Core spliceosomal proteins								
ACIN1	DDX23	HNRNPF	ISY1	PLRG1	PRPF6	SF3B2	SNRPD2	SRSF7
AQR	DHX35	HNRNPH1	LSM2	PPIE	PRPF8	SF3B3	SNRPD3	SRSF9
BCAS2	DDX41	HNRNPH3	LSM3	PPIG	PTBP1	SF3B4	SNRPE	SYF2
BUB3	DDX46	HNRNPK	LSM4	PPIH	PUF60	SLU7	SNRPF	SYNCRIP
BUD31	DHX15	HNRNPL	LSM6	PPIL1	RALY	SMNDC1	SNRPG	TXNL4A
C9orf78	DHX8	HNRNPM	LSM7	PPIL2	RBM17	SNRNP200	SNW1	U2AF1
CACTIN	EFTUD2	HNRNPR	LSM8	PPIL3	RBM22	SNRNP27	SRSF5	U2AF2
CD2BP2	FAM50A	HNRNPU	MAGOH	PPWD1	RBM8A	SNRNP40	SRP54	U2SURP
CDC40	HNRNPA0	HNRNPUL1	MATR3	PQBP1	RBMX	SNRNP70	SRRM1	USP39
CDC5L	HNRNPA1	HSPA4	NAA38	PRPF18	SART1	SNRPA	SRSF1	WBP11
CHERP	HNRNPA2B1	HSPA5	NFATC2IP	PRPF19	SF3A1	SNRPA1	SRSF12	WDR83
CRNKL1	HNRNPA3	HSPA8	NHP2L1	PRPF3	SF3A2	SNRPB	SRSF2	XAB2
CTNNBL1	HNRNPC	HTATSF1	NOSIP	PRPF31	SF3A3	SNRPB2	SRSF3	YBX1
CWC15	HNRNPD	HTRA2	PABPN1	PRPF4	SF3B1	SNRPC	SRSF4	ZMAT2
CXorf56	HNRNPDL	IK	PHF5A	PRPF4B	SF3B6	SNRPD1	SRSF6	

*Red indicates BUD31-associated protein

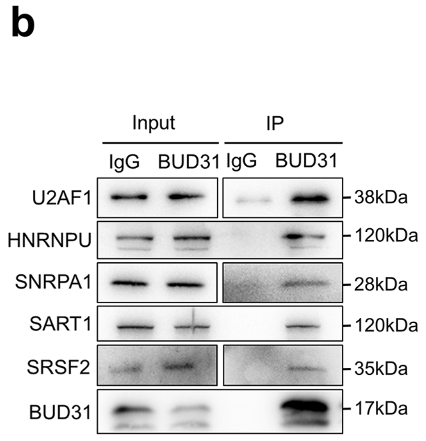

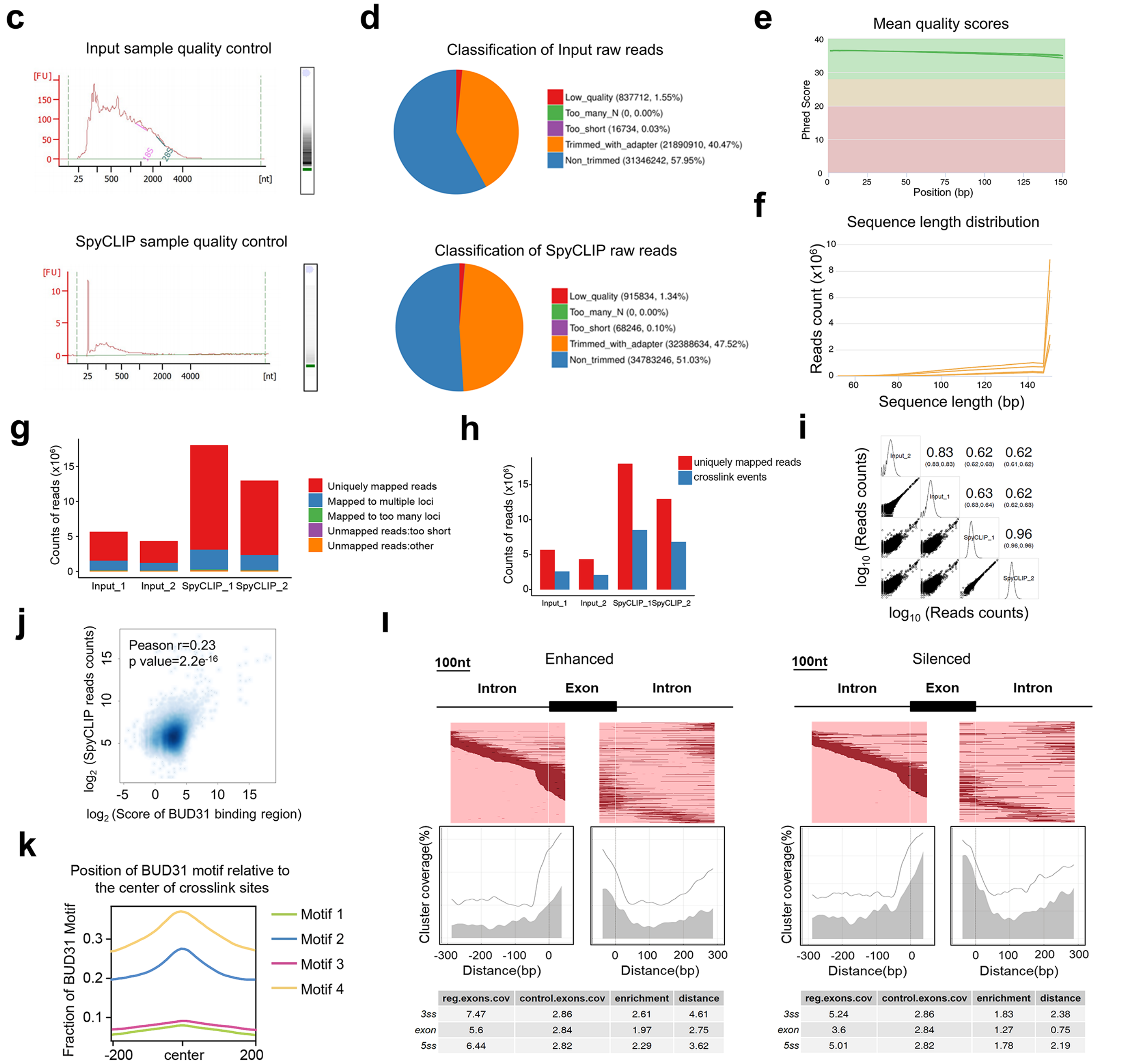

Figure. S4. BUD31 interacts with other core splicing factors and the quality control of SpyCLIP sequencing, related to Figure 4. (a) Proteins interacting with BUD31 among the 134 core splicing factors. BUD31-associated proteins resulting from IP-MS were colored in red. **(b)** Western blot analysis of co-immunoprecipitation (Co-IP) of U2AF1, HNRNPU, SNRAP1, and SART1 from HEYA8 cells with BUD31 antibody. Rabbit IgG was used as a negative control. BUD31 itself was also detected to prove efficiency. **(c)** BUD31 SpyCLIP library quality control and length distribution of Input and SpyCLIP samples examined with Agilent 2100. **(d)** Sequencing reads of Input and SpyCLIP samples. Reads with adaptors, too many N, too short reads, and low-quality reads were removed. **(e, f)** Phred means quality score **(e)** and reads length distribution **(f)** of the Input and SpyCLIP samples (n = 2 samples per group). **(g, h)** Alignment result of sequencing reads to human hg38 genome by STAR **(g)**. Uniquely mapped reads were selected and deduplicated into crosslink events **(h)**. (i) Repeatability and independence verification of SpyCLIP samples by aligning reads to the BUD31 binding regions. **(j)** Correlation between SpyCLIP reads counts and PURECLIP score of BUD31 binding regions. P-value was calculated by a two-tailed Pearson's Correlation. **(k)** Position of BUD31 motifs relative to the center of crosslink sites. 200 bp upstream and downstream was shown. **(l)** A binding map of BUD31 near the intron-exon junction of regulated alternative exons in HEYA8 cells. Each row of the heatmap shows a BUD31-regulated exon with its flanking regions. The dark red line represents the position of clusters. The enrichment of the clusters around the regulated exons compared to the control is plotted below. Left, enhanced splicing sites; right, silenced splicing sites. Source data are provided as a Source Data file.

Figure S5, related to Figure 5

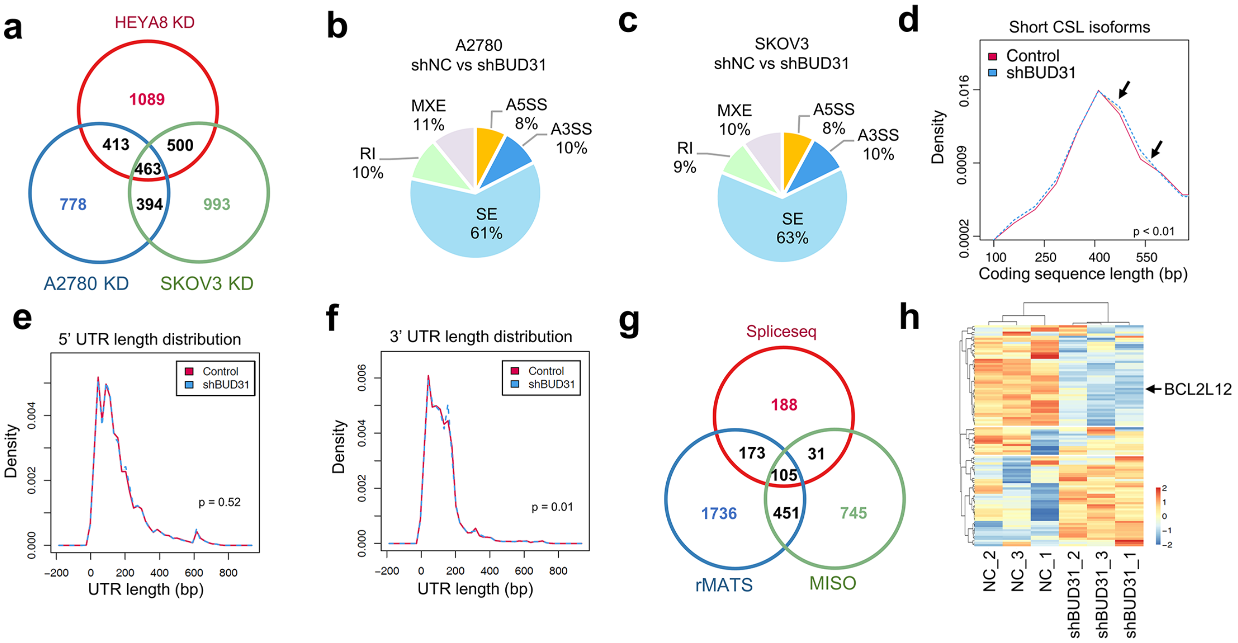

Figure. S5. BUD31 knockdown induces global alternative splicing and isoform switch, related to Figure 5. (a) AS analysis based on RNA-seq data of dox-inducible BUD31 knockdown A2780, SKOV3, and HEYA8 cells. 463 same targets were identified in the three cell lines. **(B, C)** Pie chart depicting the proportions of different types of AS events in the RNA-seq data from A2780 **(b)** and SKOV3 **(c)** cells after BUD31 knockdown. Specific differential AS events were analyzed with rMATS and were filtered out with $p < 0.05$ and |IncLevelDifference| > 0.1. SE, skipped exons; R.I., retained introns; A5SS, alternative 5' splice site; A3SS, alternative 3' splice site; MXE, mutually exclusive exons. **(d-f)** Density plot of short coding sequence **(d)**, 5' untranslated region (5' UTR) **(e),** and 3' untranslated region (3' UTR) **(f)** length distribution with silencing BUD31 in HEYA8 cells. UTR length was calculated using SpliceR with the reconstructed transcripts by Cufflinks. P-value was calculated inside the R package sm. **(g)** rMATS, Spliceseq, and MISO analyzed alternative splicing events. 2465, 497, and 1332 events were identified by the three algorithms separately, and 105 events were common to all three sets of results. **(h)** The gene expression profile of the 105 AS genes after BUD31 knockdown. The downregulated BCL2L12 was shown with a black arrow.

Figure S6, related to Figure 6 and Figure 7

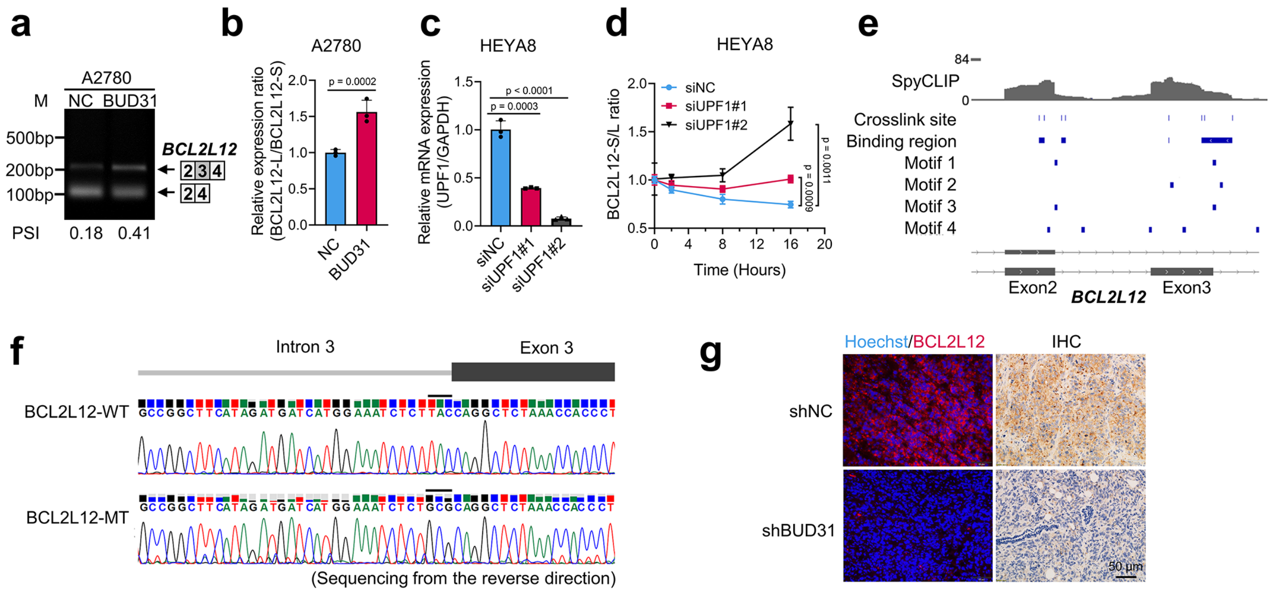

Figure. S6. *BCL2L12*-S is NMD sensitive, and BUD31 binding sites on pre-mRNA of *BCL2L12*, related to Figure 6 and Figure 7. (a) Semi-quantitative RT-PCR was performed in A2780 cells overexpression BUD31 to validate BCL2L12 AS event. **(b)** The relative transcripts expression ratio of BCL2L12-L/S after BUD31 overexpression in A2780 cells was determined by RT-qPCR. **(c)** The efficiency of 2 siRNA targeting UPF1 was determined with RT-qPCR. **(d)** The remaining *BCL2L12*-S/L mRNA ratio was calculated at 0 h, 2 h, 8 h, and 16 h after 10 μg/ml actinomycin D treatment. siUPF1 (red) was compared with siNC (blue). The residual RNAs were normalized to 0 h. **(e)** BUD31 binding motifs around the regulated exon3 of *BCL2L12*. SpyCLIP reads signal was shown in gray and crosslink sites, and BUD31 binding regions and motifs were shown in blue. **(f)** Sanger sequencing results of *BCL2L12* intron3 fragments used for RNA pull-down assay. Mutant fragment (*BCL2L12*-I3-MT) replaced TAC with GCG at the Exon3-Intron3 junction site. **(g)** BCL2L12 expression *in vivo* of the xenograft tumor with BUD31 knockdown HEYA8 cells was determined by immunofluorescence and immunohistochemistry stains. Scale bar, 50 μm. P-value was obtained by two-tailed unpaired Student's t-test **(b, c, d)**.

Figure S7, related to Figure 7

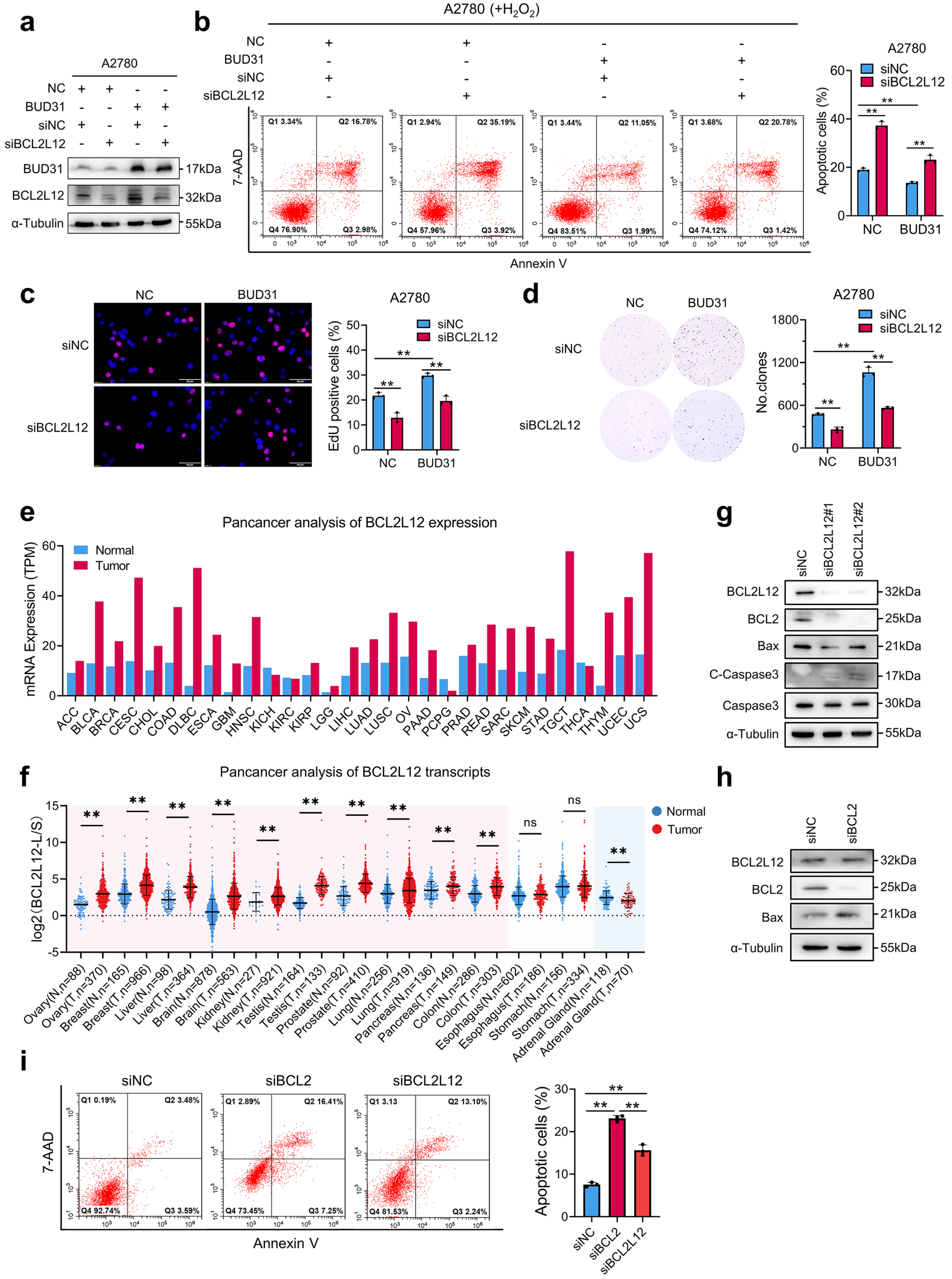

Figure. S7. BUD31 exerts its oncogenic effects in ovarian cancer by regulating BCL2L12, related to Figure 7. (a) Protein expression of BUD31 and BCL2L12 was determined with western blot in A2780 cells. **(b)** Apoptotic cells were detected by flow cytometry after BCL2L12 knockdown and BUD31 overexpression in A2780 cells. A2780 cells were treated with H_2O_2 with a final concentration of 400 μM for 4 h before apoptosis detection. **(c-d)** EdU **(c)** and clonogenic assay **(d)** indicated that knockdown BCL2L12 could attenuate the proliferation and clonogenic ability after BUD31 overexpression in A2780 cells. Scale bar, 50 μm. **(e-f)** *BCL2L12* pan-cancer expression **(e)** and *BCL2L12L/S* transcripts ratio **(f)** of tumor samples in TCGA database paired with normal tissues in Genotype-Tissue Expression Project (GTEx) database. **(g)** Western blot determined BCL2, Bax, and cleaved-caspase3 (C-Caspase3) protein expression after BCL2L12 knockdown. **(h)** Western blot determined BCL2L12 and Bax protein expression after BCL2 knockdown. **(i)** Apoptosis cell percentage of HEYA8 cells knocking down BCL2 and BCL2L12 was determined with flow cytometry. All function experiments were conducted with n = 3 biological repeats. P-value was obtained by unpaired Student's t-test, two-tailed **(b, c, d, f, i)**, and the results are presented as the mean ± S.D. *p < 0.05, **p < 0.01. Source data are provided as a Source Data file.

Figure S8, related to Figure 8

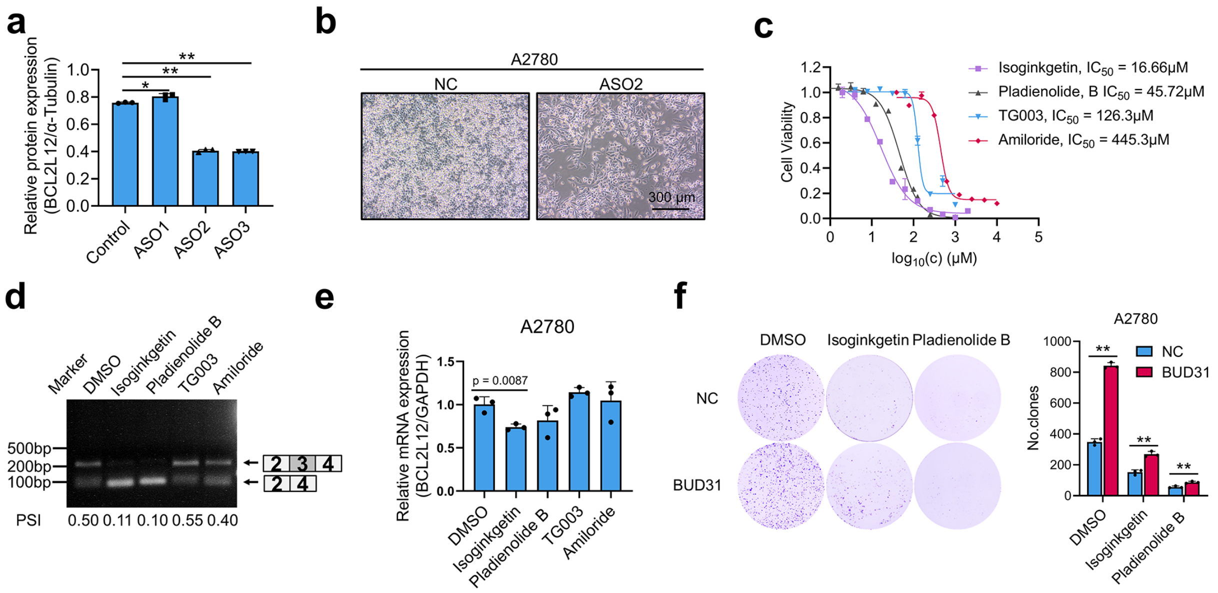

Figure. S8. ASO2 and spliceosome inhibitors inhibit cell viability and induce BCL2L12 exon 3 skipping, related to Figure 8. (a) Protein quantification of the western blot results in Figure 8c. **(b)** Cell morphology changes of A2780 cells under a microscope after 200 nM ASO2 treatment. Scale bar, 300 μm. **(c)** The viability of primary ovarian cancer cells OVBWZX treated with cisplatin, isoginkgetin (an inhibitor of U4/6-U5 snRNP), pladienolide B (a U2 snRNP inhibitor targeting SF3B1), TG003 (CLK inhibitors), and amiloride (affect splicing factor phosphorylation). **(d)** Semi-quantitative RT-PCR detected *BCL2L12* alternative splicing pattern after spliceosome inhibitors treatment. **(e)** Relative mRNA expression of *BCL2L12* was determined with RT-qPCR after inhibitors treatment. Ovarian cancer A2780 cells were treated with 100 nM Pladienolides B for 12h, 30 μM isoginkgetin for 12h,10 μM TG003 for 24h, 100 μM amiloride for 24h. **(f)** Spliceosome inhibitors isoginkgetin and pladienolide B attenuated the clonogenic ability improved by BUD31 overexpression through altering the *BCL2L12* alternative splicing pattern. All function experiments were conducted with n = 3 biological repeats. P-value was obtained by two-tailed unpaired Student's t-test **(a, c, e, f)**, and the results are presented as the mean ± S.D. *p < 0.05, **p < 0.01

Supplementary Table 1

Correlation between BUD31 expression and clinicopathological characteristics

Clinicopathological Feature	Number	BUD31 expression		*p* value
		Low	High	
**Age (years)**				
<60	103	50	53	0.0172*
≥60	46	32	14	
**FIGO Staging**				
I-II	36	23	13	0.2391
III-IV	112	59	53	
**CA 125 in serum (U/mL)**				
<500	54	28	26	0.5561
≥500	95	54	41	
**Relapse**				
No	28	20	8	0.0619
Yes	97	50	47	
**Platinum status**				
Sensitive	34	21	13	0.2273
Resistance	12	5	7	
**Lymph node metastasis**				
Present	22	10	12	0.3508
Absent	29	17	12	
**Omentum metastasis**				
Present	95	49	46	0.2099
Absent	53	33	20	

The chi-square test was used to analyze the differences in clinical characteristics. *$p < 0.05$

Supplementary Table 2 Sequence Information

Method	Name	Sequence (5′ - 3′)
**semi-PCR**	BCL2L12-E2-F	TCTCCTGTTCCAACTCCACC
**semi-PCR**	BCL2L12-E4-R	TTTCAGCTGCTCTTGGACCA
**semi-PCR**	E2F4-semiRI-F	TGTTTTGGAAGGTATCGGGC
**semi-PCR**	E2F4-semiRI-R	TGGTCTAGTTCTTGCTCCCG
**semi-PCR**	CDK16-semiRI-F	AACTACCCCAAGTACCGAGC
**semi-PCR**	CDK16-semiRI-R	AACAGCTTGGTGAGGAGGTC
**semi-PCR**	RBCK1-semi-F	ACAGGTGGCAATGAAGTGTG
**semi-PCR**	RBCK1-semi-Stop-R	CCACAGGAATTAACGGAAGAAGT
**semi-PCR**	RBCK1-semi-L-R	CTGTCATATCAGGGCGCACT
**RIP-qPCR**	BCL2L12-exon1-F	GGGTTCCTTCCCATATCCAT
**RIP-qPCR**	BCL2L12-exon1-R	ATTGAGGGAGTGGAGGGTCT
**RIP-qPCR**	BCL2L12-exon2-F	AAGACACGCTGAGGGTCCTA
**RIP-qPCR**	BCL2L12- exon2-R	GGTGGAGTTGGAACAGGAGA
**RIP-qPCR**	BCL2L12-exon3-F	CCCAAGAAGAGCCAACAGAC
**RIP-qPCR**	BCL2L12- exon3-R	CAGGGAGCAGGGAAGACATC
**RIP-qPCR**	BCL2L12- intron2-F	GGCTCCCTCCTAACTCTTGC
**RIP-qPCR**	BCL2L12- intron2-R	CCTGCAAGCAGTCTCTTTCC
**RIP-qPCR**	BCL2L12-exon2-3-F	GGTCTCCTGTTCCAACTCCA
**RIP-qPCR**	BCL2L12-exon2-3-R	CAGGGAGCAGGGAAGACAT
**RIP-qPCR**	BCL2L12-e3-i3-F	TGGTTTAGAGCCTGGTAAGAGA
**RIP-qPCR**	BCL2L12-e3-i3-R	AGCCAACCCACAGAATTGAC
**Construction**	BUD31- pTRIPZ-F	ACGAAGGGCGGAGGAGGATCCATGCCTAAAGTCAAAAGA
**Construction**	BUD31- pTRIPZ-R	TGCCGTCATAGCGCGTTAATTAATCAGCCAGAGCAGCCACG
**RNA-pulldown**	BCL2L12-E3-I3-F	TAATACGACTCACTATAGGGTGGTTTAGAGCCTGGTAAGAGA
**RNA-pulldown**	BCL2L12-E3-I3mt-F	TAATACGACTCACTATAGGGTGGTTTAGAGCCTGCGCAGAGATTTCCATGATCATCTAT
**RNA-pulldown**	BCL2L12-E3-I3-R	AGCCAACCCACAGAATTGAC
**qPCR**	BUD31-F	CATTCAGACACGGGACACCA
**qPCR**	BUD31-R	ATGATGCGGCCCACTTCC
**qPCR**	BCL2L12-F	AAGACACGCTGAGGGTCCTA
**qPCR**	BCL2L12-R	GGTGGAGTTGGAACAGGAGA
**qPCR**	BCL2L12-L-F	CCCAAGAAGAGCCAACAGAC
**qPCR**	BCL2L12-L-R	CAGGGAGCAGGGAAGACATC
**qPCR**	BCL2L12-S-F	TCCACCTAGGCCCAGCTAC
**qPCR**	BCL2L12-S-R	CGGAGATTTCAGCTGCTCTT
**qPCR**	UPF1-F	ACCTATTACACGAAGGACCTCC
**qPCR**	UPF1-R	ACGTCCGTTGCAGAACCAC
**qPCR**	BCL2 -F	GGTGGGGTCATGTGTGTGG
**qPCR**	BCL2 -R	CGGTTCAGGTACTCAGTCATCC
**qPCR**	GAPDH-F	CAGAACATCATCCCTGCCTCTAC

**qPCR**	GAPDH-R	TTGAAGTCAGAGGAGACCACCTG
**ASO design**	BCL2L12-ASO-1	C*A*G*G*C*T*C*TAAACCA*T*A*G*C*A*G
**ASO design**	BCL2L12-ASO-2	G*A*A*A*T*C*T*C*T*TACCAGG*C*T*C*T*A*A*A*C
**ASO design**	BCL2L12-ASO-3	C*A*T*A*G*A*T*GATCATG*G*A*A*A*T*C*T*C
**ASO design**	ASO-control	C*C*T*C*T*T*A*C*C*TCAGTTA*C*A*A*T*T*T*A*T*A
**siRNA sequence**	siUPF1#1	GAUGCAGUUCCGCUCCAUU
**siRNA target**	siUPF1#2	AGAGCGCATTGAAAACGTT
**siRNA target**	siBCL2L12#1	GCGGGAAAGTTGAACTAAT
**siRNA target**	siBCL2L12#2	AGCGTCACATGCAAATTGA
**siRNA target**	siCHERP	CCACCCTCATCAACGAGTA
**siRNA target**	siSRSF4	GAATCACGCTCCAGATCAA
**siRNA target**	siMATR3	GCAGGAACCTAATATGCTT
**siRNA sequence**	siSF3B4	GGAUGAGAAGGUUAGUGAATT
**siRNA sequence**	siSF3B1	GUAUUUGGGUGAAGAGUACTT
**siRNA target**	siBUD31#1	GGAGTTGATTGAGCCAACA
**siRNA target**	siBUD31#2	CTGGATGAATTAGATCAAA
**siRNA target**	siBUD31#3	TCTTCGACCTCTTTTACAA
**siRNA target**	siRBCK1	GCGTGTTATGCACTCAATAATAA
**siRNA target**	siBCL2	GGAGAACAGGGTACGATAA
**siRNA sequence**	siNC	UUCUCCGAACGUGUCACGUTT

*: indicated phosphorothioate modifications.

Supplementary Table 3 Key resources table

REAGENT or RESOURCE	SOURCE	IDENTIFIER
Antibodies		
Rabbit polyclonal anti-BUD31	Proteintech	11798-1-AP; RRID: AB_2274894
Mouse monoclonal anti-alpha-Tubulin	Proteintech	66031-1-Ig; RRID: AB_11042766
Rabbit polyclonal anti-SF3B1	Proteintech	27684-1-AP; RRID: AB_2880946
Rabbit polyclonal anti-PARP1	Proteintech	13371-1-AP; RRID: AB_2160459
Rabbit polyclonal anti-Cleaved-Caspase3	Cell Signaling Technology	#9661; RRID: AB_2341188
Rabbit polyclonal anti-Caspase3	Cell Signaling Technology	#9662; RRID: AB_331439
Rabbit monoclonal anti-SC35	Abcam	ab204916; RRID: AB_2909393
Rabbit polyclonal anti-Bax	Affinity	AF0120; RRID: AB_2833304
Mouse monoclonal anti-BCL2	Origene	TA803003; RRID: AB_2626627
Rabbit polyclonal anti-U2AF1	ABclonal	A6076; RRID: AB_2766739
Rabbit polyclonal anti-HNRNPU	ABclonal	A3917; RRID: AB_2765383
Rabbit polyclonal anti-SART1	ABclonal	A8569; RRID: AB_2772144
Rabbit polyclonal anti-SNRPA1	ABclonal	A12161; RRID: AB_2759048
Rabbit monoclonal anti-BCL2L12	Abways	CY9733
Rabbit monoclonal anti-E2F4	Zen bioscience	R24161
Rabbit monoclonal anti-CDK16	Zen bioscience	R26239
Mouse monoclonal anti-HuR	Thermo Scientific	1862775
Rabbit polyclonal anti-GAPDH	Zen bioscience	380626
Bacterial and virus strains		
Escherichia coli BL21(DE3) competent cells	Vazyme	C504-02
Escherichia coli DH5α competent cells	Vazyme	C502-02
Biological samples		

HGSOC specimens and fallopian tube tissues	Department of Obstetrics and Gynecology, Qilu Hospital	N/A
Ascites-derived ovarian cancer cells from patients	Department of Obstetrics and Gynecology, Qilu Hospital	N/A
Chemicals, peptides, and recombinant proteins		
Isopropyl β-D-thiogalactoside	BBI	A600168
Penicillin-Streptomycin Solution, 100X	Biosharp	BL505A
Sodium pyruvate	Sigma	P4562
Insulin	Macgene	CC101
Dimethyl sulfoxide (DMSO)	Aladding	D103277
Doxycycline hydrochloride	BBI	A600889
D-Luciferin, Sodium Salt	Yeason	40901ES01
Chloral hydrate	Sangon	A600288
Cisplatin	Sigma	PHR1624
Pladienolides B	Santa Cruz	sc-391691
Isoginkgetin	MedChemExpress	HY-N2117
TG003	MedChemExpress	HY-15338
Amiloride hydrochloride	Targetmol	T0175
Crystal violet	Solarbio	C8470
Trypsin without ethylenediaminetetraacetic acid (EDTA)	Macgene	CC035
Critical commercial assays		
His-tag Protein Purification Kit	Beyotime	P2226
Cell Total RNA Isolation Kit	Foregene	RE-03111
Cell-Light EdU Apollo567 In Vitro Kit	RiboBio	C10310-1
RNAMAX-T7 in vitro transcription kit	RiboBio	C11001-1
Annexin V-PE/7-AAD Apoptosis Detection Kit	Vazyme	A213-01
TUNEL Cell Apoptosis Detection Kit	KEYGEN	KGA703
Pierce™ RNA 3’ End Desthiobiotinylation Kit	Thermo Fisher Scientific	20163
Magnetic RNA Protein Pull-Down Kit	Thermo Fisher Scientific	20164
CoolShift-BTr RNA EMSA Kit	Viagene	SIDET102
Spin Column RNA Cleanup & Concentration Kit	Sangon	B518688
Nucleoprotein Extraction Kit	Sangon	C500009
EZ-Nuclear RIP Kit	Merck Millipore	17-701
Deposited data		
RNA sequencing (RNA-seq) of BUD31 knockdown by shRNA in ovarian cancer cells	This paper	GEO: GSE183449
RNA immunoprecipitation sequencing (RIP-seq) of BUD31 in ovarian cancer cells	This paper	GEO: GSE183450

SpyCLIP sequencing of BUD31 in ovarian cancer cells	This paper	GEO: GSE183451
Experimental models: Cell lines		
Human: A2780 ovarian cancer cell line	Jian-Jun Wei' lab	N/A
Human: HEY ovarian cancer cell line	Jian-Jun Wei' lab	N/A
Human: OV90 ovarian cancer cell line	ATCC	CRL-11732
Human: OVCAR3 ovarian cancer cell line	ATCC	HTB-161
Mouse: ID8 Ovarian Surface Epithelial Cell Line	Sigma-Aldrich	SCC145
Experimental models: Organisms/strains		
Mouse: CAnN.Cg-Foxn1nu/CrlVr	Vitalriver	BALB/c-nude mice
Oligonucleotides		
See Supplementary Table 2	This paper	N/A
Recombinant DNA		
Doxycycline-inducible lentiviral vector pTRIPZ-NC	Zhao et al., 2019 (PMID: 30715466)	N/A
SpyCatcher-pET-28a	Zhao et al., 2019 (PMID: 30715466)	N/A
pZIP-TRE3G-shBUD31	Transomic	TLHSU2300
pENTER-BUD31	WZ Biosciences	CH803738
pENTER-BCL2L12	WZ Biosciences	CH801321
Software and algorithms		
FastQC v0.11.9	N/A	https://www.bioinformatics.babraham.ac.uk/projects/fastqc/
Trimgalore v0.6.1	N/A	https://www.bioinformatics.babraham.ac.uk/projects/trim_galore/
bowtie v1.2.3	Langmead et al., 2009 (PMID: 19261174)	http://bowtie-bio.sourceforge.net/index.shtml
STAR v2.7.1a	Dobin et al., 2013 (PMID: 23104886)	https://github.com/alexdobin/STAR
HISAT2 v2.2.0	Zhang et al., 2021 (PMID: 34103331)	http://daehwankimlab.github.io/hisat2/
Salmon v0.6.0	Patro et al., 2017 (PMID: 28263959)	https://combine-lab.github.io/salmon/

Picard v2.25.5	N/A	https://gatk.broadinstitute.org/hc/en-us/articles/360057439771-MarkDuplicates-Picard-
Qualimap v2.2.2	García-Alcalde et al., 2012 (PMID: 22914218)	http://qualimap.conesalab.org/
Samtools v1.10	Li et al., 2009 (PMID: 19505943)	http://samtools.sourceforge.net/
StringTie v1.3.0	Kovaka S et al., 2019 (PMID: 31842956)	http://ccb.jhu.edu/software/stringtie/
featureCounts v2.0.0	Liao Y et al., 2014 (Liao Y et al., 2014)	http://subread.sourceforge.net/featureCounts.html
rMATS v4.1.0	Shen et al., 2014 (PMID: 25480548)	http://rnaseq-mats.sourceforge.net/
ASprofile	Florea L et al., 2013 (PMID: 24555089)	http://ccb.jhu.edu/software/ASprofile/
MISO (Mixture of Isoforms) v0.5.4	Katz et al., 2010 (PMID: 21057496)	https://miso.readthedocs.io/en/fastmiso/
TCGASpliceseq	Ryan M et al.,2016 (PMID: 26602693)	https://bioinformatics.mdanderson.org/TCGASpliceSeq/
IsoformSwitchAnalyzeR v3.13	Vitting-Seerup et al., 2019 (PMID: 30989184)	https://github.com/kvittingseerup/IsoformSwitchAnalyzeR
Coding Potential Assessment Tool (CPAT) v1.2.1	Wang et al., 2013 (PMID: 23335781)	http://lilab.research.bcm.edu/
Pfam v34.0	Mistry et al., 2021 (PMID: 33125078)	https://pfam.xfam.org/
Signalp v5.0b	Almagro Armenteros et al., 2019 (PMID: 30778233)	http://www.cbs.dtu.dk/services/SignalP/
SpliceR v1.14.0	Vitting-Seerup et al., 2014 (PMID: 24655717)	http://www.bioconductor.org/packages/2.13/bioc/html/spliceR.html

edgeR v3.34.0	Robinson et al., 2010 (PMID: 19910308)	https://bioconductor.org/packages/release/bioc/html/edgeR.html
Survival v3.2-11	N/A	https://cran.r-project.org/web/packages/survival/index.html
Survminer v0.4.9	N/A	https:// github.com/ kassambara/ survminer/
ggplot2 v3.3.5	N/A	https://ggplot2.tidyverse.org
PURECLIP v1.2.0	Krakau et al., 2017 (PMID: 29284540)	https://github.com/skrakau/PureCLIP
HOMER v4.11	Heinz et al., 2010 (PMID: 20513432)	http://homer.ucsd.edu/homer/
deeptools v3.1.3	N/A	https://github.com/jernejule/clip-data-science.
UCSC Xena	Goldman et al., 2020 (PMID: 32444850)	https://xenabrowser.net
PANTHER	N/A	http://pantherdb.org/
Gene Ontology	The Gene Ontology, 2019 (PMID: 30395331)	http://geneontology.org/
Gene Set Enrichment Analysis	Subramanian et al., 2005 (PMID: 16199517)	https://www.gsea-msigdb.org/gsea
Kaplan-Meier plotter	N/A	http://kmplot.com/analysis/
R/Bioconductor v3.6.3	N/A	http://www.r-project.org/
GraphPad Prism 8	N/A	https://www.graphpad.com/scientific-software/prism/
ImageJ v2.1.0	Schneider et al., 2012 (PMID: 22930834)	https://imagej.nih.gov/ij/
Integrative Genomics Viewer (IGV) v2.8.2	Robinson et al., 2017 (PMID: 29092934)	https://igv.org/
Other		

Code used to analyze data and generate figures	This study	https://github.com/PrinceWang2018/BUD31_BCL2L12
Visualization of binding regions distributions around the regulated exons	Anob et al., 2017 (doi: 10.1101/208124)	https://github.com/jernejule/clip-data-science.
