# Supplementary material for: Splicing factor BUD31 promotes ovarian cancer progression through sustaining the expression of anti-apoptotic BCL2L12
Source: Nat Commun. 2022 Oct 21;13:6246. doi: 10.1038/s41467-022-34042-w (PMC9587234; doi:10.1038/s41467-022-34042-w)
Supplement: Supplementary file 3 — Description of Additional Supplementary Files [file 41467_2022_34042_MOESM3_ESM.pdf]

Description of Additional Supplementary Files

File Name: Supplementary Data 1
Description: Differentially expressed splicing factors and prognostic analysis

File Name: Supplementary Data 2
Description: BUD31 interacting proteins identified by IP-MS

File Name: Supplementary Data 3
Description: Target genes of BUD31
