# Supplementary material for: Splicing factor BUD31 promotes ovarian cancer progression through sustaining the expression of anti-apoptotic BCL2L12
Source: Nat Commun. 2022 Oct 21;13:6246. doi: 10.1038/s41467-022-34042-w (PMC9587234; doi:10.1038/s41467-022-34042-w)
Supplement: Supplementary file 7 — Reporting Summary [file 41467_2022_34042_MOESM7_ESM.pdf]

nature portfolio

Corresponding author(s): Zhaojian Liu

Last updated by author(s): Sep 28, 2022

Reporting Summary

Nature Portfolio wishes to improve the reproducibility of the work that we publish. This form provides structure for consistency and transparency in reporting. For further information on Nature Portfolio policies, see our Editorial Policies and the Editorial Policy Checklist.

Statistics

For all statistical analyses, confirm that the following items are present in the figure legend, table legend, main text, or Methods section.

n/a	Confirmed	
☐	☒	The exact sample size (*n*) for each experimental group/condition, given as a discrete number and unit of measurement
☐	☒	A statement on whether measurements were taken from distinct samples or whether the same sample was measured repeatedly
☐	☒	The statistical test(s) used AND whether they are one- or two-sided<br>*Only common tests should be described solely by name; describe more complex techniques in the Methods section.*
☒	☐	A description of all covariates tested
☐	☒	A description of any assumptions or corrections, such as tests of normality and adjustment for multiple comparisons
☐	☒	A full description of the statistical parameters including central tendency (e.g. means) or other basic estimates (e.g. regression coefficient) AND variation (e.g. standard deviation) or associated estimates of uncertainty (e.g. confidence intervals)
☐	☒	For null hypothesis testing, the test statistic (e.g. *F*, *t*, *r*) with confidence intervals, effect sizes, degrees of freedom and *P* value noted<br>*Give P values as exact values whenever suitable.*
☒	☐	For Bayesian analysis, information on the choice of priors and Markov chain Monte Carlo settings
☒	☐	For hierarchical and complex designs, identification of the appropriate level for tests and full reporting of outcomes
☐	☒	Estimates of effect sizes (e.g. Cohen's *d*, Pearson's *r*), indicating how they were calculated

Our web collection on statistics for biologists contains articles on many of the points above.

Software and code

Policy information about availability of computer code

Data collection	QuantStudio Design & Analysis software (version 1.4.2) was used for qPCR data.<br>CytExpert (version 2.4) was used to collect flow cytometry data.<br>Spy-CLIP was sequenced by paired-end 150bp sequencing performed on the Illumina HiSeq 2500 platform.<br>RNA-seq data was generated by sequencing performed on the Illumina HiSeq 4000 platform.
Data analysis	Western blot and agarose gel electrophoresis quantification and colony number counting: Fiji (version 1.53c).<br>Statistical analysis: R/R Bioconductor 3.6.3 and GraphPad Prism (version 8).<br>RNA-seq reads were aligned to the human genome (hg38) with HISAT2 (version 2.2.0). Alternative splicing was quantified using rMATS (version 4.1.0), and MISO (Mixture of Isoforms, version 0.5.4). Transcripts were reconstructed with StringTie (version 1.3.0). Differential gene expression analysis was conducted using edgeR (version 3.36.0). Transcript expression and isoform switch were determined using Salmon (version 0.6.0) and IsoformSwitchAnalyzeR (version 3.13).<br>Spy-CLIP data processing was conducted using the previously described iCLIP analysis pipeline (Busch, A, et al. 2020). The adaptor sequence and low-quality reads were removed with TrimGalore (version 0.6.1), and the quality of the clean reads was checked with FastQC (version 0.11.9). rRNA sequences were removed with bowtie (version 1.2.3) . The remaining reads were mapped to the human genome (hg38) using the STAR software (version 2.7.1a) . PCR duplicates of uniquely mapped reads were removed using Picard (version 2.25.5) with MarkDuplicates. IGV browser (version 2.6.2) was used for CLIP-seq visualization. Crosslink sites were identified with PURECLIP (version 1.2.0). Annotation and binding motif identification were conducted using HOMER (version 4.11). Motifs were matched to the genome position with scanMotifGenomeWide.pl function belonging to HOMER (version 4.11) and visualized with deeptools (version 3.1.3).<br>The custom code used to analyze the data has been deposited at https://github.com/PrinceWang2018/BUD31_BCL2L12. The analysis pipelines are publicly available as of the date of publication.

For manuscripts utilizing custom algorithms or software that are central to the research but not yet described in published literature, software must be made available to editors and reviewers. We strongly encourage code deposition in a community repository (e.g. GitHub). See the Nature Portfolio guidelines for submitting code & software for further information.

Data

Policy information about availability of data

All manuscripts must include a data availability statement. This statement should provide the following information, where applicable:

- Accession codes, unique identifiers, or web links for publicly available datasets
- A description of any restrictions on data availability
- For clinical datasets or third party data, please ensure that the statement adheres to our policy

The gene expression profiles were obtained from the TCGA (https://portal.gdc.cancer.gov/) and GTEx (https://www.gtexportal.org/) databases. The protein expression profiles were obtained from the CPTAC database (https://proteomics.cancer.gov/programs/cptac). Exon expression and isoform percentage were viewed and downloaded from UCSC Xena (https://xenabrowser.net/). PSI values of AS events in ovarian cancer were collected from TCGASpliceseq (http://bioinformatics.mdanderson.org/TCGASpliceSeq). An online Kaplan–Meier plotter database (http://kmplot.com/analysis/) was used in the prognostic analysis.
The RNA-seq, RIP-seq, and SpyCLIP data for this study are available for download from the Gene Expression Omnibus (GEO) repository (GSE183449, GSE183450, and GSE183451) . All the three datasets were summarized and deposited at GEO database (GSE183452). The following secure token has been created to allow review of record GSE183452: mbehmskuzpirbyx. The raw data for IP-MS are available in Table S3. The data are publicly available as of the date of publication.

Field-specific reporting

Please select the one below that is the best fit for your research. If you are not sure, read the appropriate sections before making your selection.

☒ Life sciences ☐ Behavioural & social sciences ☐ Ecological, evolutionary & environmental sciences

For a reference copy of the document with all sections, see nature.com/documents/nr-reporting-summary-flat.pdf

Life sciences study design

All studies must disclose on these points even when the disclosure is negative.

Sample size	No statistical methods were used to pre-determine sample size. The sample size of each experiment is provided in the figure/table legends in the main manuscript and supplementary file. For RNA-seq, samples were prepared in three biological replicates. For SpyCLIP-seq, samples were prepared at least in two biological replicates according to previous reported experiment design (Zhao Y, et al. 2019). For in vivo mouse model, each group has at least 6 mice. For experiments with live cells, each plot was repeated at least twice with similar results. These sizes have previously been shown as sufficiently powered to determine statistical differences in mean values of our investigated parameters.
Data exclusions	No data was excluded from analysis, except for necessary data quality control step for initial RNA-seq and CLIP-seq data processing.
Replication	The replication number is indicated in the legend of corresponding figures where applicable.
Randomization	Cells and mice were randomly allocated into experimental groups. Randomized grouping is conducted using a computer program to generate pseudo-random numbers.
Blinding	All the control and experimental group of mice/cells were grown under identical conditions. The investigators were not blinded as proper controls were already included during experiment design.

Reporting for specific materials, systems and methods

We require information from authors about some types of materials, experimental systems and methods used in many studies. Here, indicate whether each material, system or method listed is relevant to your study. If you are not sure if a list item applies to your research, read the appropriate section before selecting a response.

Materials & experimental systems

n/a	Involved in the study	
☐	☒	Antibodies
☐	☒	Eukaryotic cell lines
☒	☐	Palaeontology and archaeology
☐	☒	Animals and other organisms
☐	☒	Human research participants
☒	☐	Clinical data
☒	☐	Dual use research of concern

Methods

n/a	Involved in the study	
☒	☐	ChIP-seq
☐	☒	Flow cytometry
☒	☐	MRI-based neuroimaging

Antibodies

Antibodies used	Mouse monoclonal anti-alpha tubulin Proteintech Cat: 66031-1-Ig; RRID: AB_11042766

Antibodies used	Rabbit polyclonal anti-BUD31 Proteintech Cat: 11798-1-AP; RRID: AB_2274894<br>Rabbit polyclonal anti-SF3B1 Proteintech Cat: 27684-1-AP; RRID: AB_2880946<br>Rabbit polyclonal anti-PARP1 Proteintech Cat: 13371-1-AP; RRID: AB_2160459<br>Rabbit polyclonal anti-Cleaved-Caspase3 Cell Signaling Technology Cat: #9661; RRID: AB_2341188<br>Rabbit polyclonal anti-Caspase3 Cell Signaling Technology Cat: #9662; RRID: AB_331439<br>Rabbit monoclonal anti-SC35 Abcam Cat: ab204916; RRID: AB_2909393<br>Rabbit polyclonal anti-Bax Affinity Cat: AB_2833304; RRID: AB_2833304<br>Mouse monoclonal anti-BCL2 Origene Cat: TA803003; RRID: AB_2626627<br>Rabbit polyclonal anti-U2AF1 ABclonal Cat: A6076; RRID: AB_2766739<br>Rabbit polyclonal anti-HNRNPU ABclonal Cat: A3917; RRID: AB_2765383<br>Rabbit polyclonal anti-SART1 ABclonal Cat: A8569; RRID: AB_2772144<br>Rabbit polyclonal anti-SNRPA1 ABclonal Cat: A12161; RRID: AB_2759048<br>Rabbit monoclonal anti-BCL2L12 Abways Cat: CY9733<br>Rabbit monoclonal anti-E2F4 Zen bioscience Cat: R24161<br>Rabbit monoclonal anti-CDK16 Zen bioscience Cat: R26239<br>Mouse monoclonal anti-HuR Thermo Scientific Cat: 1862775<br>Rabbit polyclonal anti-GAPDH Zen bioscience Cat: 380626<br>Peroxidase AffiniPure Goat Anti-Human IgG (H+L) Jackson ImmunoResearch Cat: 109-035-003<br>Peroxidase AffiniPure Goat Anti-Mouse IgG (H+L) Jackson ImmunoResearch Cat: 115-035-003
Validation	All the antibodies tested in the manuscript has been published or described previously. Validation and citation information can be found on the manufacturer's website. Their original links are listed in the below:<br>Mouse monoclonal anti-alpha-Tubulin WB,IF: https://www.ptgcn.com/products/tubulin-Alpha-Antibody-66031-1-Ig.htm<br>Rabbit polyclonal anti-BUD31 WB,IF,IHC: https://www.ptgcn.com/products/BUD31-Antibody-11798-1-AP.htm<br>Rabbit polyclonal anti-SF3B1 WB: https://www.ptgcn.com/products/SF3B1-Antibody-27684-1-AP.htm<br>Rabbit polyclonal anti-PARP1 WB: https://www.ptgcn.com/products/PARP1-Antibody-13371-1-AP.htm<br>Rabbit polyclonal anti-Cleaved-Caspase3 WB: https://www.cellsignal.com/products/primary-antibodies/cleaved-caspase-3-asp175-antibody/9661<br>Rabbit polyclonal anti-Caspase3 WB: https://www.cellsignal.com/products/primary-antibodies/caspase-3-antibody/9662<br>Rabbit monoclonal anti-SC35 IF: https://www.abcam.com/sc35-antibody-epr12238-ab204916.html<br>Rabbit polyclonal anti-Bax WB: http://affbiotech.cn/goods-45-AF0120-Bax_Antibody.html<br>Mouse monoclonal anti-BCL2 WB: https://www.origene.com/catalog/antibodies/primary-antibodies/ta803003/bcl2-mouse-monoclonal-antibody-clone-id-oti2e5<br>Rabbit polyclonal anti-U2AF1 WB: https://abclonal.com.cn/catalog/A6076<br>Rabbit polyclonal anti-HNRNPU WB: https://abclonal.com.cn/catalog/A3917<br>Rabbit polyclonal anti-SART1 WB: https://ap.abclonal.com/catalog-antibodies/SART1RabbitpAb/A8569<br>Rabbit polyclonal anti-SNRPA1 WB: https://abclonal.com.cn/catalog/A12161<br>Rabbit monoclonal anti-BCL2L12 WB,IF,IHC: http://www.abways.cn/showproduct.asp?cid=CY9733<br>Rabbit monoclonal anti-E2F4 WB: http://www.zen-bio.cn/prod_view.aspx?IsActiveTarget=True&TypeId=167&Id=523066&FId=t3:167:3<br>Rabbit monoclonal anti-CDK16 WB: http://www.zen-bio.cn/prod_view.aspx?IsActiveTarget=True&TypeId=167&Id=524596&FId=t3:167:3<br>Mouse monoclonal anti-HuR WB: https://assets.thermofisher.cn/TFS-Assets/LSG/SDS/1862775_MTR-APLT_CN.pdf<br>Rabbit polyclonal anti-GAPDH WB: http://www.zen-bio.cn/prod_view.aspx?IsActiveTarget=True&TypeId=147&Id=505658&FId=t3:147:3

Eukaryotic cell lines

Policy information about cell lines

Cell line source(s)	Human ovarian cancer cell lines A2780 and HEYA8 were obtained from the Jian-Jun Wei lab, Northwestern University. HEK293T cell line was obtained from the National Collection of Authenticated Cell Cultures. OV90 and OVCAR3 cell lines were purchased from the American Type Culture Collection. The mouse ovarian surface epithelial cell line ID8 was purchased from Sigma-Aldrich (SCC145).
Authentication	Cell lines were validated by STR profiling.
Mycoplasma contamination	All cell lines tested negative for mycoplasma contamination.
Commonly misidentified lines (See ICLAC register)	None.

Animals and other organisms

Policy information about studies involving animals; ARRIVE guidelines recommended for reporting animal research

Laboratory animals	In the subcutaneous xenograft model, female BALB/c-nude mice (6–8 weeks old) (Vital River Laboratory Animal Technology) were used. Less than 6 mice with same sex were housed in a cage at 20-25 °C and 50% humidity with a 12 h light/dark cycle.
Wild animals	None
Field-collected samples	None
Ethics oversight	The Shandong University Animal Ethics Research Board approved the animal experiment procedures (SDULCLL2019-2-08).

Note that full information on the approval of the study protocol must also be provided in the manuscript.

Human research participants

Policy information about studies involving human research participants

Population characteristics	Patient ascites was collected from the Department of Obstetrics and Gynecology, Qilu Hospital, Shandong University, upon the patient's informed consent. Ascites-derived ovarian cancer cells OVBWZX were obtained from a 54 years old female patient diagnosed with high-grade serous ovarian cancer by clinical pathology before receiving chemotherapy and surgical therapy. Primary ovarian cancer cells were cultured according to the previous studies (Shepherd TG, 2006).
Recruitment	The patient was recruited after clinical diagnosis with high-grade serous ovarian cancer and ascites. The ascites was collected before receiving chemotherapy and surgical therapy. It was collected from one patient and the study did not involve potential self-selection bias.
Ethics oversight	The patient provided informed consent, and ethical approval was granted by the Ethics Committee of Shandong University (SDULCLL2019-1-09).

Note that full information on the approval of the study protocol must also be provided in the manuscript.

Flow Cytometry

Plots

Confirm that:

☒ The axis labels state the marker and fluorochrome used (e.g. CD4-FITC).

☒ The axis scales are clearly visible. Include numbers along axes only for bottom left plot of group (a 'group' is an analysis of identical markers).

☒ All plots are contour plots with outliers or pseudocolor plots.

☒ A numerical value for number of cells or percentage (with statistics) is provided.

Methodology

Sample preparation	Flow cytometry analysis for cell apoptosis was performed using an Annexin V-PE/7-AAD Apoptosis Detection Kit (Vazyme, A213-01). In H2O2-induced apoptosis experiments, cells were treated with H2O2 with a final concentration of 400 μM for 4 h before apoptosis detection. Cells were digested with ethylenediaminetetraacetic acid (EDTA)-free trypsin (Macgene, CC035) for 3 min, collected by centrifugation, washed with ice-cold phosphate-buffered saline (PBS) and resuspended at a density of 5 × 105 cells/ml with 100 μl 1× Binding Buffer. Then 5 μl Annexin V-PE and 5 μl 7-AAD were added and incubated for 10 min in the dark. Finally, cells were incubated with an additional 400 μl 1× Binding Buffer and analyzed within 20 min by CytoFLEX S (Beckman Coulter Life Science).
Instrument	CytoFLEX S (Beckman Coulter Life Science)
Software	CytExpert version 2.4
Cell population abundance	At least 1 × 104 cells were analyzed to determine the percentage of apoptotic cells. The purity of the ovarian cancer cell line samples used in vitro was satisfactory.
Gating strategy	The starting cell population was selected through FSC/SCC gates. Debris and dead cells with a lower level of forward scatter and are found at the bottom left corner of the density plot. The forward scatter threshold was increased to avoid collecting these events. The density plot is split into four quadrants allowing to determine the cells single positive for Annexin V-PE or 7-AAD and both double negative and double positive. The relative proportion of early or late apoptosis cells were quantified by placing gates around the distinct populations.

☒ Tick this box to confirm that a figure exemplifying the gating strategy is provided in the Supplementary Information.
